# Immune suppressed tumor microenvironment by exosomes derived from gastric cancer cells via modulating immune functions

**DOI:** 10.1038/s41598-020-71573-y

**Published:** 2020-09-08

**Authors:** Juan Liu, Shaoxian Wu, Xiao Zheng, Panpan Zheng, Yuanyuan Fu, Changping Wu, Binfeng Lu, Jingfang Ju, Jingting Jiang

**Affiliations:** 1 Department of Tumor Biological Treatment, The Third Affiliated Hospital of Soochow University, Changzhou, 213003 China; 2 Jiangsu Engineering Research Center for Tumor Immunotherapy, Changzhou, 213003 China; 3 Institute of Cell Therapy, Soochow University, Changzhou, 213003 China; 4 Department of Pathology, Stony Brook University, New York, 11794 USA; 5 Department of Immunology, University of Pittsburgh, Pittsburgh, 15213 USA

**Keywords:** Tumour immunology, Immunology

## Abstract

Gastric cancer is one of the leading causes of cancer-related death due to late diagnosis with high metastatic frequency. In this study, the impact of tumor secreted exosomes on immune function in the tumor environment was investigated using exosomes isolated from gastric cancer cell lines MKN-28, MKN-45, and SGC-7901. Results show that exosomes derived from all of these cell lines changed the gene expression and cytokine secretion levels of CD8^+^ T cells. They also block cell cycle progression, induced apoptosis in CD8^+^ T cells. Image analysis of fluorescent labeled exosomes derived from three cell lines injected systemically into C57BL/6 mice revealed these exosomes primarily localize to the lungs. We further showed exosomes were mainly taken up by natural killer cells and macrophages in the lung. After long-term exposure to inject exosomes from MKN-45 cells, mice developed an immunosuppressive tumor microenvironment in the lung with increased frequency of effector memory CD4^+^ T and MDSC, decreased CD8^+^ T cell and NK frequency. This immune suppressive environment promotes gastric cancer lung metastasis. Lung metastasis sites developed after mice were exposed to exosomes isolated from all three gastric cancer cell lines when the mice were injected with MFC cells. Results suggest that exosomes derived from gastric cancer cells (especially MKN-45 and MKN-28) changed CD8^+^ T cell gene expression and cytokine secretion patterns to create an immunosuppressive condition for metastatic niche formation in the lung. Overall, this study provides new insights into how gastric cancer derived exosomes modulate the immune response to promote lung tumor metastasis.

## Introduction

Gastric cancer is one of the most common malignancies worldwide^1,2^. Recent projections suggest that there will be 27,600 new cases and 11,010 deaths from gastric cancer in the United States alone in 2020^3^. Metastasis to distant sites is the most common cause of cancer death. Patients with gastric cancer exhibit high incidence in metastasis rate, mortality rate, and low early diagnosis rate^4–6^. The majority of incidence and mortality occur in developing countries^4^. Loo et al*.*^7^ found that certain molecules in the tumor microenvironment are important components and targets of tumor malignant catalysis. Recent studies have shown that tumor cells secrete a broad spectrum of bioactive molecules^8^ to modulate the immune response^9,10^. One way in which tumor cells modulate the immune environment is through the release of extracellular vesicles. Tumor cells release different types of extracellular vesicles in body fluids^11^. Based on their biogenesis, these extracellular vesicles are divided into three main classes exosomes, mircovesicles and apoptotic bodies^12^. Exosomes are enclosed by a phospholipid lipid biolayer membrane with a flat or spherical shape and a diameter ranging from 30 to 150 nm^13^. Exosomes contain multiple biologically active molecules such as DNA, RNA, protein and lipid^14^. It has been shown that small membranous particles contributed significantly to intercellular communication and reprogramming of the tumor microenvironment^15–17^. This reprograming can help cancer cells evade the immune system. Exsomes may also play a role in promoting metastatic niche formation. The molecular mechanism underlying this immune evasion and metastatic niche formation remains unclear.

Studies have reported that exosome secrection levels are correlated with tumor stage and metastasis^18,19^. Studies have revealed that tumor derived exosomes play a crucial role in remodeling the tumor microenvironment immune profile through suppressing specific T cell immunity and educate innate immune cells towards a pro-tumor phenotype^20–22^. The key mechanism is to reduce immune surveillance for primary tumors by creating a permissive environment in the development of the metastatic niche^23^. In the crosstalk between the tumor and its surrounded microenvironment, exosomes exert multiple functions in shaping the tumor immune response^24,25^. Tumor escape from the host immune system has been considered an obstacle to biotherapy. CD8^+^ T cells act as a central player in tumor regression^26^. It is believed that melanoma derived exosomes can interact with CD8^+^ T cells^9^, and studies have found that prostate cancer derived exosomes induce T cell apoptosis and dysfunction^27^, there are other findings that tumor derived exosomes promote CD8^+^ T activation and differentiation^28^.

In general, poorly differentiated gastric cancer released more exosome, whether from human samples or from established gastric cancer cell lines^29^. Based on this, we reasoned that differentiation status of gastric cancer may influence metastasis by secreting unique exosomes to create an immunosuppressive microenvironment. In gastric cancer, it remains to be determined whether gastric cancer exosomes will induce CD8^+^ T cell dysfunction and promote metastatic niche formation. In this study, we focus on the impact and mechanisms of gastric cancer exosomes on lung metastasis. By using gastric cancer-derived exosomes with different differentiation status (well differentiated gastric cancer cell line MKN-28, poorly differentiated gastric cancer cell line MKN-45, and moderately differentiated gastric cancer cell line SGC-7901), we investigated the effect of gastric cancer exosomes on CD8^+^ T cells by specifically focusing on cell cycle progression, apoptosis, cytokine secretion, and gene expression. The biodistribution of exosomes and their impact on the immune subpopulation were investigated in vivo after injection of exosomes from these three gastric cancer cell lines into mice via tail vein. Our results show that gastric cancer exosomes promote pre-metastic niche formation and produce a immunosuppressive microenvironment by influencing the immune subpopultion under long-term exosome exposure conditions.

## Result

### Characterization of gastric cancer derived exosomes

**Figure 1:**
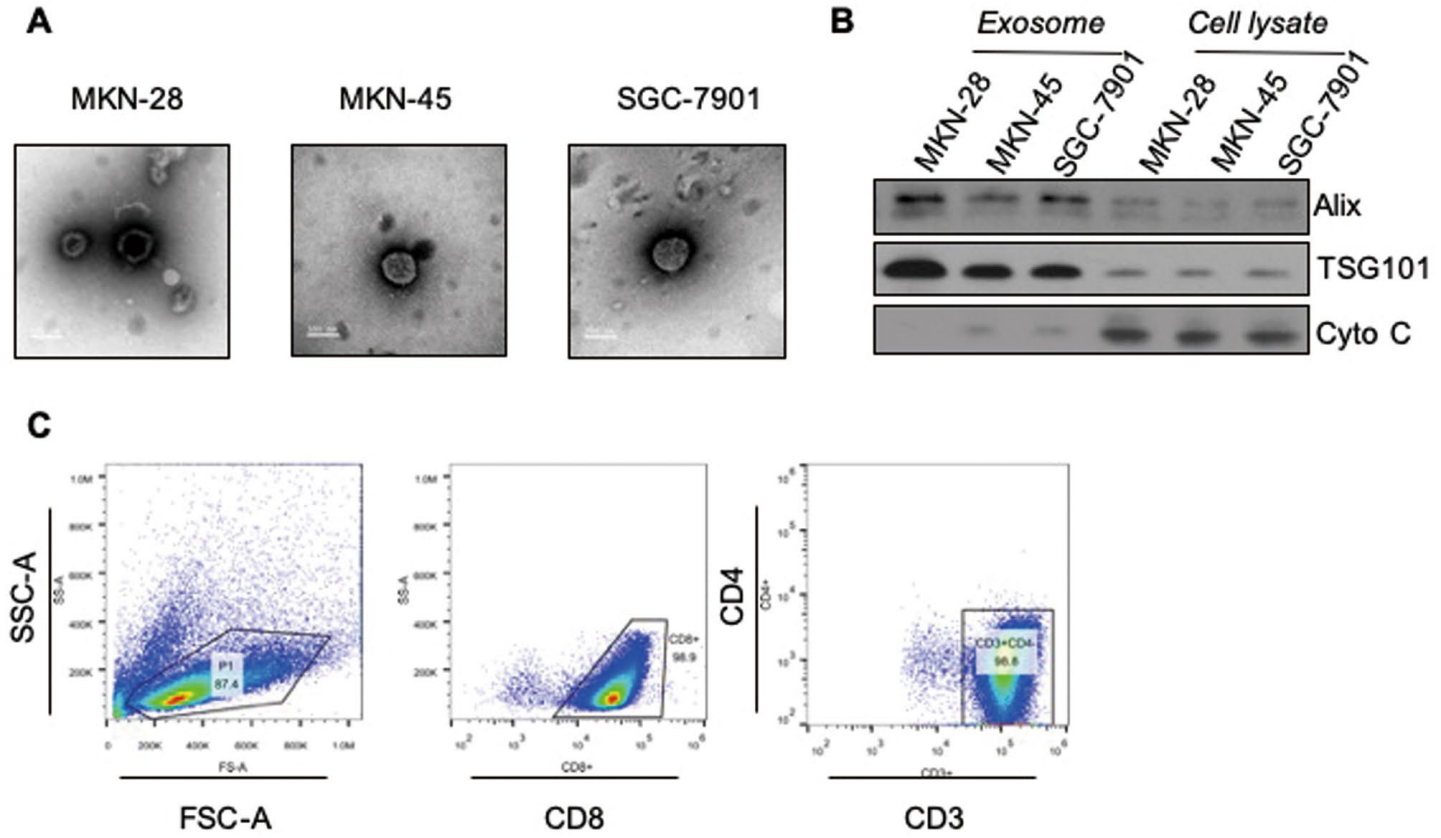
Identification of gastric cancer-derived exosomes and CD8^+^ T cell. **(A)** Morphological characterization of exosome. The exosome vesicle structure was visible under an electron microscope with a diameter of 30–150 nm. **(B)** Western blot showed that the extracted material expressed exosome molecular markers Alix and TSG101. **(C)** Isolated CD8^+^ T cell subset was with CD3^+^CD8^+^CD4^-^ phenotype, and the purity of CD8^+^ T cells was over 98%.

Exosomes were successfully extracted from gastric cancer cell lines (MKN-28, MKN-45, and SGC-7901) via ultracentrifugation. Electron microscopy was performed to assess the spherical shape and size of the purified exosomes. The exosome vesicle structure was visualized by TEM with a diameter ranging from 30–150 nm (Fig. 1A). To further confirm that the material indeed consisted of purified exosome, Western immunoblot analysis was performed to show the expression of exosome specific markers Alix and Tsg101 (Fig. 1B).

### Effects of gastric cancer derived exosomes on apoptosis of CD8^+^ T cells

CD8^+^ T cells were isolated from normal donor’s PBMC with highly enriched yield and subset of cells, which were identified as the subset of cells that are CD3^+^CD8^+^CD4^-^ phenotype with purity over 98% by flow cytometry analysis (Fig. 1C).

**Figure 2:**
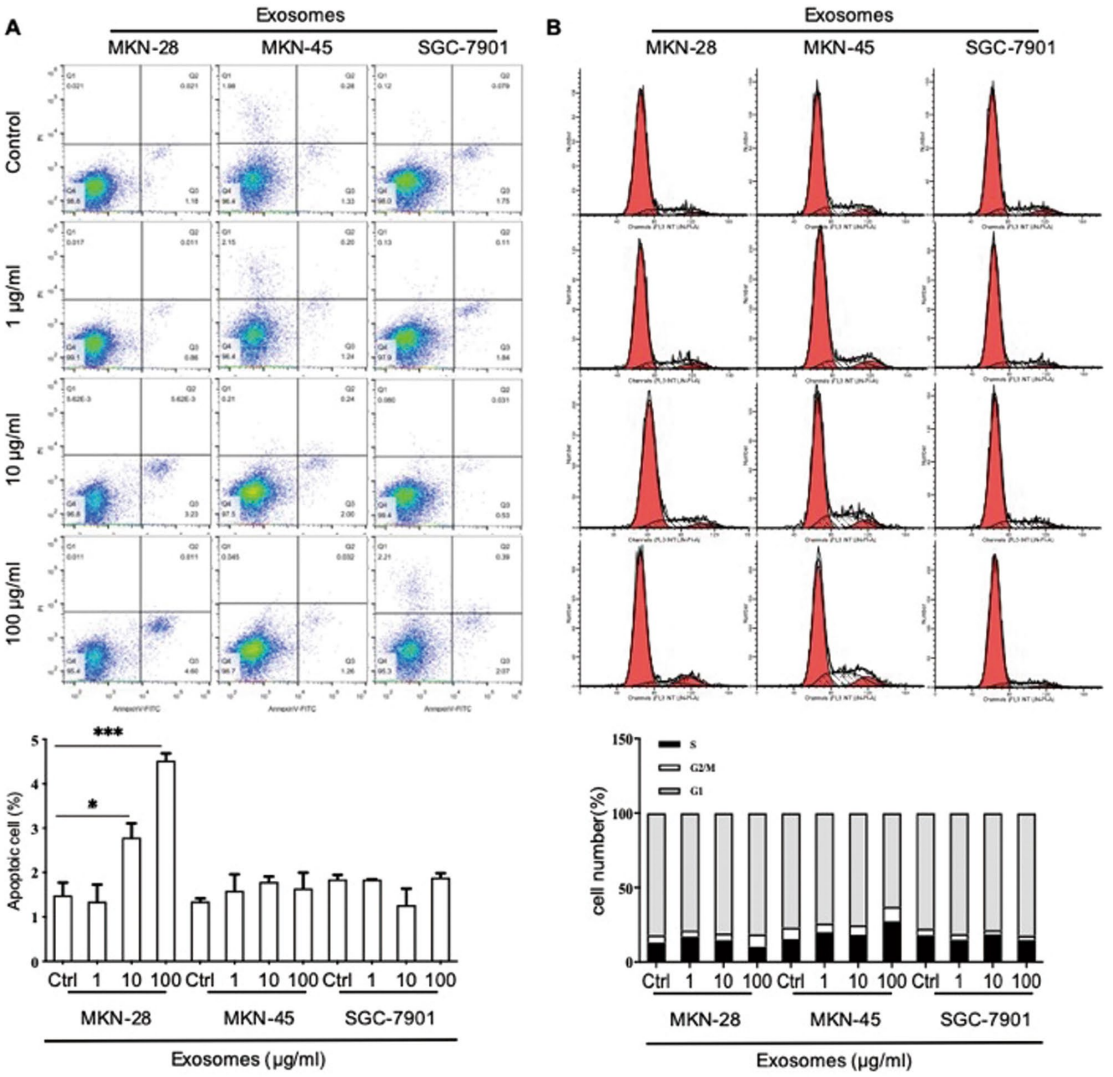
The function of exosome from SGC-7901, MKN-45, MKN-28 cell lines on CD8^+^ T cell apoptosis and cell cycle in vitro. (**A**) Flow cytometry detected CD8^+^ T cell apoptosis after stimulation with exosomes from three gastric cancer cell lines. (**B**) Cell cycle analysis of CD8^+^ T cells after stimulated by exosomes from three gastric cancer cell lines. Results are presented as mean ± SEM and analyzed by ANOVA. *, *P* < 0.05, **, *P* < 0.01, ***, *P* < 0.001.

We were interested to determine if gastric cancer cell line derived exosomes induce apoptosis of CD8^+^ T cells. We incubated CD8^+^ T cells with exosomes derived from MKN-28, MKN-45 and SGC-7901 gastric cancer cells for 48 h. MKN-28 derived exosomes induced apoptosis of CD8^+^ T cells at 10 and 100 μg/ml in a dose dependent manner (2.79 ± 0.55 vs 1.49 ± 0.49, *P* = 0.036; 4.52 ± 0.28 vs 1.49 ± 0.49, *P* < 0.0001, Fig. 2A). Exosomes derived from MKN-45 or SGC-7901 gastric cancer cells had no effect on CD8^+^ T cells apoptosis (*P* > 0.05, Fig. 2A). These results suggest that exosomes from gastric cancer cell lines with different differentiation status did not induce a major change in apoptosis of CD8^+^ T cells.

### Gastric cancer-derived exosomes alter the cell cycle of CD8^+^ T cells

To determine the effect of gastric cancer derived exosomes on the cell cycle progression of CD8^+^ T cells, we incubated CD8^+^ T cells with various concentrations of exosomes derived from three gastric cancer cell lines (MKN-28, MKN-45, SGC-7901). The percent of CD8^+^ T cells in G2 phase was significantly increased after stimulation with 100 μg/ml MKN-28 derived exosomes for 48 h (8.25 ± 1.46 *vs* 4.93 ± 1.75, *P* = 0.032). After treated with 100 μg/ml MKN-45 derived exosomes, the proportion of CD8^+^ T cells in G1 phase decreased (62.84 ± 2.22 *vs* 76.93 ± 3.03, *P* = 0.014), and the proportion in S phase increased (27.19 ± 0.90 *vs* 15.14 ± 2.46, *P* = 0.000). After stimulation with 100 μg/ml SGC-7901 derived exosomes, the proportion of CD8^+^ T cells in G2 phase was decreased (2.87 ± 0.21 *vs* 3.81 ± 0.55, *P* = 0.016). There was no significant difference in the cell cycle of CD8^+^ T cells incubated with lower concentrations of gastric cancer derived exosomes (1 and 10 μg/ml, *P* > 0.05, Fig. 2B). This result show that the cell cycle of CD8^+^ T cells was affected by exosomes from gastric cancer cell lines only at 100 μg/ml.

### Effect of gastric cancer exosomes on CD8^+^ T cytokine secretion

**Figure 3:**
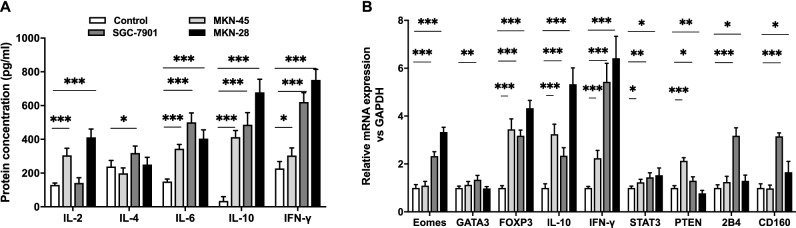
Gastric cancer exosome altered CD8^+^ T cell cytokine secretion and gene expression. (**A**) ELISA analysis of IL-2, IL-4, IL-6, IL-10, and IFN-γ expression level with or without SGC-7901, MKN-45, MKN-28 derived exosomes. (**B**) Real-time qPCR analysis of Eomes, FOXP3, IL-10, IFN-γ, 2B4, CD160, GATA3, STAT3, PTEN gene expression with or without SGC-7901, MKN-45, MKN-28 derived exosomes. Results are presented as mean ± SEM and analyzed by ANOVA. *, *P* < 0.05, **, *P* < 0.01, ***, *P* < 0.001.

We also tested the effects of gastric cancer derived exosomes on cytokine production by CD8^+^ T cells. We found that production of the cytokines tested (IL-2, IL-4, IL-6, IL-10, IFN-γ) were up-regulated after exposure to gastric cancer derived exosomes. Compared with control group, 100 μg/ml MKN-45 derived exosomes stimulated CD8^+^ T cells to increase secretion of IL-2, IL-6, IL-10, and IFN-γ (*P* < 0.05). IL-10 expression was increased by 12.26 times in CD8^+^ T cells. While, IL-2, IL-6, and IFN-γ showed only slight upregulation of approximately two fold. IL-4 secretion was not altered compared to control group. Exosomes derived from SGC-7901 at 100 μg/ml stimulated the secretion of IL-4, IL-6, IL-10, and IFN-γ by CD8^+^ T cells (*P* < 0.05). IL-10 expression was increased 13.82 fold. Exosomes derived from MKN-28 at 100 μg/ml stimulated CD8^+^ T cell secretion of IL-2, IL-6, IL-10 and IFN-γ (*P* < 0.05). The expression of IL-10 secreted by CD8^+^ T cells stimulated with 100 μg/ml MKN-28 derived exosomes was 19.53 times higher than control group. The effect of MKN-28 derived exosomes on secretion of cytokines by CD8^+^ T cells was significantly different from those derived from SGC-7901 and MKN-45 (Fig. 3A). All tested cytokine was upregulation compared with control group. The result show that gastric cancer derived exosomes stimulate secretion of immune suppressive cytokine IL-10 by CD8^+^ T cells. These results suggest that exosomes from gastric cancer cell lines may impact tumor microenvironment by increasing immunosuppressive cytokine secretion by CD8^+^ T cells.

### Effect of gastric cancer exosomes on CD8^+^ T cell gene expression

Our results show that expression levels of eomesodermin (Eomes), forkhead box protein P3 (FOXP3), interleukin 10 (IL-10), interferon gamma (IFN-γ), CD244 molecule (2B4), CD160 molecule (CD160), GATA binding protein 3(GATA3), signal transducer and activator of transcription 3 (STAT3), and phosphatase and tensin homolog (PTEN) were significantly upregulated in CD8^+^ T cells exposed to exosomes derived from three gastric cancer cell line comparison with control group. CD8^+^ T cells stimulated by MKN-45 derived exosomes expressed FOXP3, IL-10, IFN-γ, PTEN and STAT3 at higher levels (*P* < 0.05). FOXP3 expression was increased 3.46 fold. SGC-7901 derived exosome stimulated CD8^+^ T cells had increased expression of Eomes, FOXP3, IL-10, IFN-γ, 2B4, CD160, GATA3, STAT3, and PTEN (*P* < 0.05). IFN-γ and FOXP3 expression were increased 5.44 and 3.19 times in CD8^+^ T cells compared to control group, respectively. MKN-28 derived exosomes increased the expression of Eomes, FOXP3, IL-10, IFN-γ, STAT3, PTEN, CD160, and 2B4 in CD8^+^ T cells (*P* < 0.05). FOXP3 was increased 4.35 fold, IL-10 was increased 5.40 fold and IFN-γ was upregulated 6.43 fold. Gene expression of CD8^+^ T cell stimulated by MKN-28 derived exosome had significantly increased FOXP3, IL-10 and IFN-γ compared with another groups (Fig. 3B). The results demonstrate that the immune suppressive gene FOXP3 and IL-10 were highly expressed in CD8^+^ T cells after exposed to exosomes from gastric cancer cells, which suggest that exosomes from gastric cancer are required to promote an immunosuppresive phenotype.

### Biodistribution of gastric cancer derived exosomes in vivo

To elucidate the role of gastric cancer derived exosomes in intercellular communication and its target effects, we investigated the biodistribution of different differentiation status gastric cancer exosomes in syngeneic mice after systemic delivery. We examined the tissue distribution of exosomes derived from MKN-45, SGC-7901, and MKN-28 to assess whether different differentiate status of gastric cancer cells can influence the uptake and the distribution of tumor exosomes. The distribution of exosomes was assessed at 4 h and 48 h after intravenous injection using flourescence imaging in C57BL/6 mice. MKN-28, MKN-45, and SGC-7901 derived exosomes were primarily accumulated in the lung within 4 h. By 48 h, MKN-45 derived exosomes were significantly distributed in the kidney, liver, and bone marrow, partial residues still existed in the lung. SGC-7901 derived exosomes were significantly present in the kidney, no obvious signal was seen in other organs. MKN-28 derived exosomes mainly remained in the lung (Supplementary Figure S1). These results support the notion that differentiation status of gastric cancer cells impacts the distribution of their secrected exosomes.

### Uptake and effect by immune cell subpopulation of gastric cancer derived exosomes in vivo

**Figure 4:**
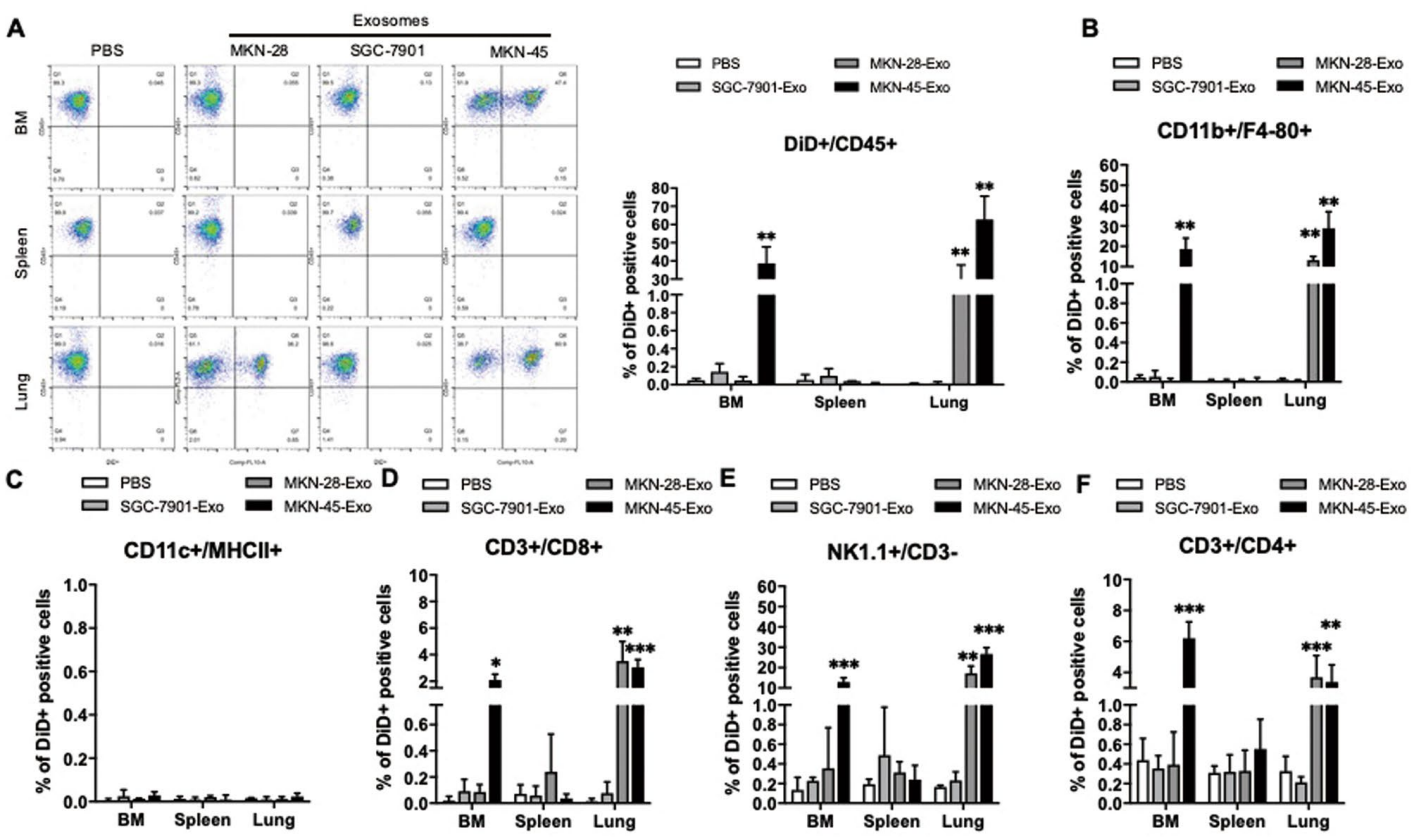
Uptake of SGC-7901, MKN-45, MKN-28 derived exosomes by immune cell subpopulation. The percentage fluorescent signal of exosome uptake by immune populations in the bone marrow BM, spleen, and lung was analyzed post 48 h by flow cytometry after injection of DiD-labeled SGC-7901, MKN-45, MKN-28-derived exosomes (20 μg/mice) or PBS (control) in C57BL/6 mice. (**A**) Representative flow cytometric plots (left) and quantification (right) of distinct DiD^+^ population within CD45^+^ cells. (**B–F**)**,** Frequency of the DiD^+^ subpopulation within macrophages (Mø; CD11b^+^/F4/80^+^, **B**), DCs (CD11C^+^/MHCII^+^, **C**), CD8 T cells (CD3^+^/CD8^+^, **D**), NK cells (NK1.1^+^/CD3^−^, **E**) and CD4 T cells (CD3^+^/CD4^+^, **F**). Results are presented as mean ± SEM and analyzed by ANOVA, *, *P* < 0.05, **, *P* < 0.01, ***, *P* < 0.001.

In vivo quantification of fluorescent signal shows significant accumulation in the lung within 4 h after injection. After 48 h, this signal was decreased in the lung, but was detectable in other organs. Next, to determine the cell lineage that were taking up the fluorescent-labeled exosomes from three different gastric cancer cell lines, we choose lung, bone marrow and spleen tissues to obtain CD45^+^ cells and verified whether exosomes were taken up by CD45^+^ cells and separated immune cell subpopulations to analyze the immune microenvironment. Our results show that exosomes derived from SGC-7901 cells were hardly absorbed by CD45^+^ immune cells in the bone marrow, spleen and lung. Exosomes from MKN-28 cells were mainly absorbed by CD45^+^ immune cells in the lung, while exosomes from poorly differentiated MKN-45 cells were taken up by CD45^+^ immune cells with high intensity in the bone marrow and lung (Fig. 4A).

Further study showed within each CD45^+^ cell subpopulation, that exosomes derived from SGC-7901 was hardly absorbed by CD45^+^ cell and subpopulation immune cells in the bone marrow, spleen, and lung. Exosomes from MKN-28 cells were distributed in M_φ_ (CD11b^+^/F4/80^+^, Fig. 4B), CD8^+^ (CD3^+^/CD8^+^, Fig. 4D), NK (NK1.1^+^/CD3^-^, Fig. 4E) and CD4^+^ (CD3^+^/CD4^+^, Fig. 4F) cells, were mainly taken up by NK and M_φ_ in the lung. While the poorly differentiated MKN-45 exosomes were mainly distributed in NK and M_φ_ of the lung and bone marrow. Exosomes from poorly differentiated MKN-45 cells were taken up by CD4^+^ and CD8^+^ T cells was much less than NK and M_φ_ in the bone marrow and lung. Exosomes from all three gastric cancer cell lines were not taken up by DC (CD11C^+^/MHCII^+^, Fig. 4C). These results indicate that exosomes derived from gastric cancer cells maybe skew the immune system by altering NK and M_φ_.

### Systemic immunosuppressive modulatory effect induced by gastric cancer exosome

**Figure 5:**
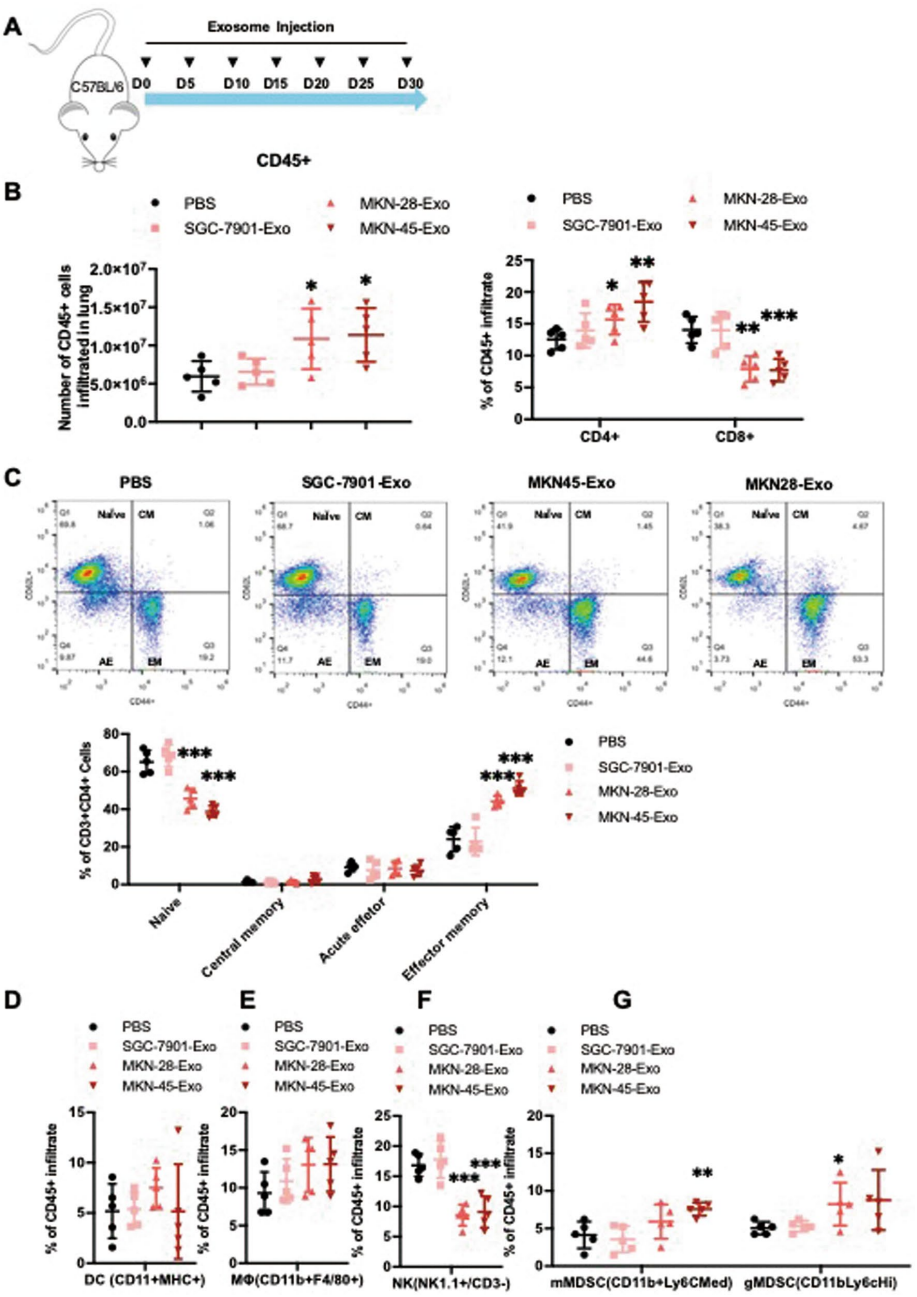
Gastric cancer derived exosomes promote an immunosuppressive premetastatic niche formation in the lung. (**A**) Twenty C57BL/6 mice were divided into four groups. Mice in each group were injected with 20 μg SGC-7901, MKN-45, MKN-28 derived exosomes and PBS every 5 days, for a total of 30 days (tail intravenous). After 30 days, the organs were harvested, and the immune cells were obtained. Flow cytometry quantified the frequencies of immune subpopulations in the lungs of mice in each group. **(B)** Frequencies of subpopulations of CD4 (CD3^+^/CD4^+^) and CD8 T cells (CD3^+^/CD8^+^) subpopulations. **(C)** Representative frequencies of subpopulations of (naïve (CD44^low^/CD62L^high^), central memory (CM, CD44^high^/CD62L^high^), effector memory (EM, CD44^high^/CD62L^low^), and acute effector CD4^+^T cells (AE, CD44^low^/CD62L^low^) in the lung. **(D–G)** Representative frequency of DCs (CD11C^+^/MHCII^+^, **D**). Macrophages (Mø, CD11b^+^/F4/80^+^, **E**), NK (NK1.1^+^/CD3^−^, **F**) and MDSCs (gMDSCs, CD11b + /Ly6C^med^, mMDSCs CD11b^+^/Ly6C^high^, **G**), respectively. Results are presented as mean ± SEM (*n* = 5 animals/group) and analyzed by two-tailed Mann–Whitney *U* tests, *, *P* < 0.05, **, *P* < 0.01, ***, *P* < 0.001.

To further examine the role of gastric cancer-derived exosomes in conditioning the lung to potentially create a premetastatic niche, exosomes derived from three gastric cancer cell lines were injected into C57BL/6 mice every 5 days for 30 days (Fig. 5A). Subsequence characterization of infiltration immune cells in the lung confirmed significant changes in immune composition. Long-term injection of exosomes derived SGC-7901 had no impact on the infiltrating immune cell in the lung. However, exosomes derived from MKN-28 and MKN-45 both significantly induced an immunosuppressive microenvironment in the lung. Results shown that the frequency of CD8^+^ T cell decreased while CD4^+^ T cell frequency increased (Fig. 5B). Further analysis of CD4^+^ T cell subset to show that naïve T cells were decreased, and effective memory T cells were increased in the MKN-45 and MKN-28 derived exosome treated groups (Fig. 5C). Next we analyzed CD45^+^ immune cell subset to show that NK cell frequency was decreased (Fig. 5F), M_φ_ and MDSC population frequency were increased (Fig. 5E, G) in the MKN-45 derived exosome treated group. While exosomes from all three gastric cancer cell lines were not absorbed by DC (Fig. 5D). Overall, our results suggest that exosomes from a poorly differentiated gastric cancer cell line MKN-45 are more capable than that of MKN-28 and SGC-7901 cells to induce CD4^+^ T cell with CD62^low^CD44^hi^ phenotype and recruiting of MDSC.

**Figure 6:**
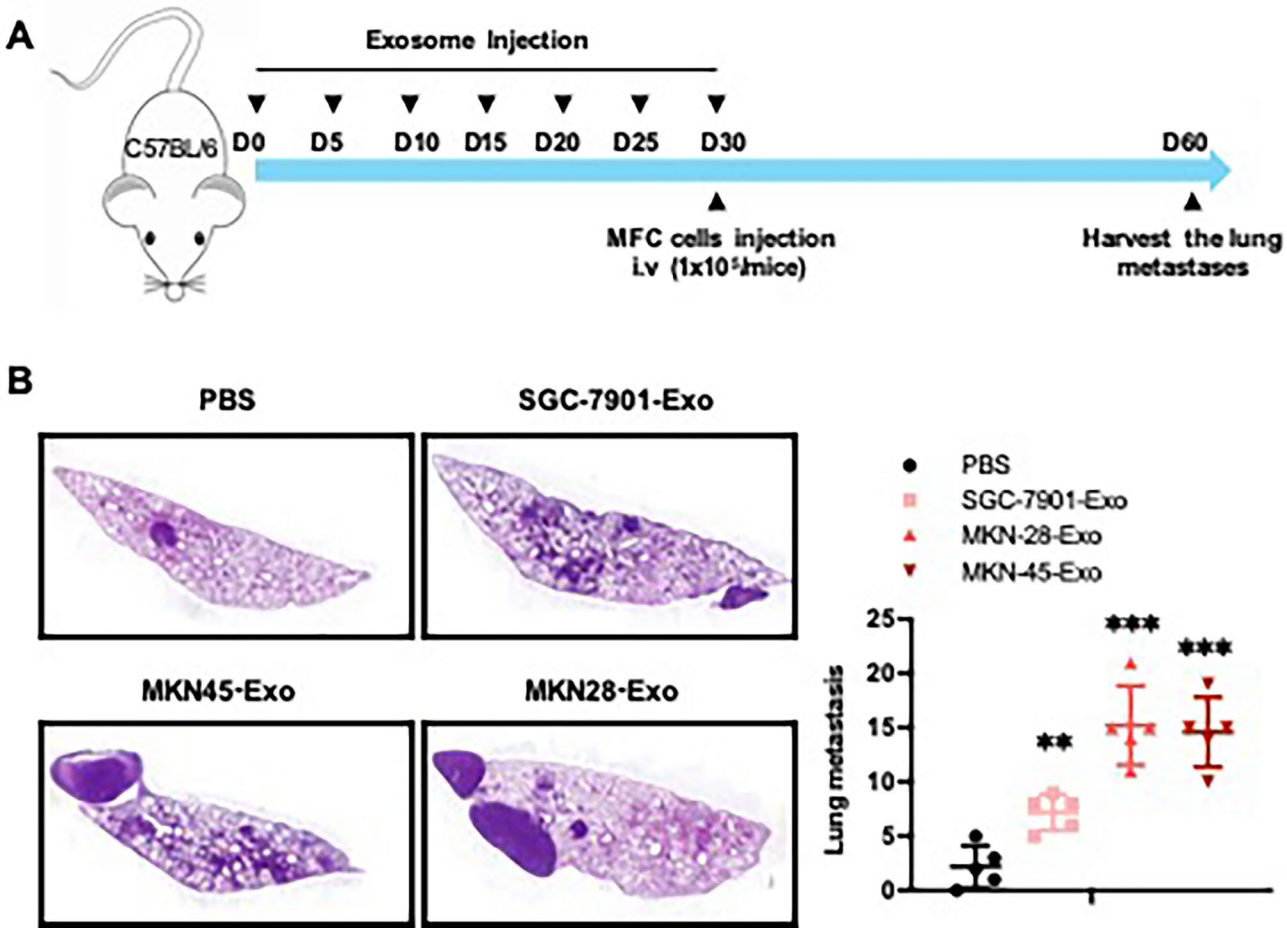
Gastric cancer derived exosomes promote distant metastasis in the lung. (**A**). C57BL/6 mice received 1 × 10^5^ MFC cells and metastatic sites in the lung were assessed 30 days later after continues conditioning injection of SGC-7901, MKN-45, MKN-28-derived exosomes, or PBS every 5 days for 30 days (tail intravenous). (**B**)**.** Identification of lung metastases by hematoxylin and eosin staining (circled in black). Number of metastases sites per lung section (two sections per lung analyzed). Results are presented as mean ± SEM (*n* = 5 animals/group) and analyzed by ANOVA. **, *P* < 0.01, ***, *P* < 0.001.

To determine the overall immunosuppressive microenvironment created by exosomes derived from gastric cancer cell lines. Following the 30 days conditioning of naïve mice with exosomes derived MKN-28, MKN-45 and SGC-7901, C57BL/6 mice received 1 × 10^5^ MFC (Mouse Forestomach Carcinoma) cells via tail vein (Fig. 6A). Long-term injection of exosomes secreted by MKN-28, MKN-45 and SGC-7901cells increased the number of MFC cells in lung metastases. After long-term stimulation with exosomes derived from MKN-28 and MKN-45 cells, the lung metastatic burden was significantly higher than that of exosomes derived from SGC-7901 cells (Fig. 6B). These results suggest that exosomes from poorly differentiated MKN-45 cells had a larger impact on creating a pre-metastatic niche to promote metastasis.

## Discussion

In this study, we investigated the impact of exosomes on tumor microenvironment using purified exosomes from three gastric cancer cell lines with different differentiation status. We found that exosomes derived from gastric cancer cells significantly triggered cell cycle arrest, changed the gene expression and cytokine secretion patern of CD8^+^ T cells within 48 h while triggered limited amount of apoptosis. Exosome are an important mediator of cell-to-cell communication and mediate multiple physiological and pathological processes. Studies have shown that exosomes from melanoma cells carried immune suppressive molecules (such as Programmed cell death 1 ligand 1, PD-L1) that interacted with CD8^+^ T cells inhibing their function^9^. Previous studies have reported that the distribution of exosomes is dependent on their parental cells^30^. In experiments reported by others, human primary CD8^+^ T cells incubated with tumor derived exosomes had altered gene expression levels and cytokine secretion patterns^31^ and showed that exosomes inhibited CD3-ζ and Janus kinase 2 (JAK) expression and suppress the follow-up T-cell receptor (TCR) signaling^32,33^. Exosomes also express bioactive FasL and selectively induce T cell apoptosis via Fas cell surface death receptor/ Fas cell surface death receptor ligand (Fas/FasL) interaction^22,27,34^. Co-expression of IL-10 and FOXP3 effectively medicated immunosuppression. Our results show that gastric cancer derived exosome can impair immune function by increasing suppressive cytokine secretion and suppressive gene to induce cell dysfunction which inducate that gastric cancer derived exosomes represents a mechanism by which tumor cells escape from the host immune system.

Here, we show that continuous conditioning of the lung with exosomes from MKN-45 gastric cancer cells in vivo increased differentiation of naïve T cell to effecter T cell, a phenotype observed in other tumor microenvironments where T cells are pushed to terminal differentiation to exhaust T cells^35^. When effector cytotoxic T cells enter tumor microenvironment, they encounter a complicated network of cells and cytokines that suppress their effectiveness and turn them into exhausted T cells. Exhausted T cells are no longer functional with up-regulated expression of inhibitory receptors, decreased production of effective cytokines, and reduced cytotoxic activity leading to cancer immune evasion^36^. In this study, exosomes derived from MKN-28, MKN-45 and SGC-7901demonstrated different potential in their impact on the immune environment both in vitro and in vivo. Further studies are needed to explore how exosomes interact with immune cell and cellular uptake, as well as identifying the key molecule(s) in the exosome that impacting the target cell.

We reasoned that exosome derived from gastric cancer may be responsible for inducing an immunosuppression tumor microenvironment. To study the exosome derived from gastric cancer function in vivo, we used a model system to create an immunosuppressive microenvironment via exosome derived from gastric cancer. Our study shows that fluorescent labeled exosomes initially accumulated mainly in the mouse lung^37^, and to a lesser extend in spleen and bone marrow. The information on biodistribution will be essential to understand gastric cancer derived exosomes on the impact of gastric cancer metastasis.

Evading immune surveillance during metastasis and sustained growth is dependent on multiple immunosuppressive surrounding cell populations^38^. We found that up to 62.77% CD45^+^ cells in the lung took up exosomes from MKN-45 gastric cancer cells, which suggests that the highly metastatic nature of poorly differentiated gastric cancer cell MKN-45 may pre-regulate the immune microenvironment of metastatic niche via releasing exosome^37^. The majority of exosomes derived from MKN-45 cells were uptaken by M_φ_ and NK cells, which may indicate poorly differentiated MKN-45 exosomes pre-regulated NK and M_φ_ in the immune microenvironment of metastatic niche thereby altering their recognition and killing capibility on tumor cells. However, it is unclear from this study if exosome uptake is cell specific. In addition, target specificity of immune cells for binding of exosome may be determined by adhesion molecules such as tetraspanins protein, integrins, as well as major histocompatibility complex class (MHC) present on exosome, and proteins with selective target combination of surface marker^39^. Increasing evidence show that tumor derived exosomes induce tumor immune escape by impairing the function of immune cells, incuding T cells, NK, DC, through the enrichment of some biological proteins such as PD-L1 or galectin-9 on exosome in different cancer^10,40^. Future studies are needed to determine the specific uptake by different immune cell subsets and whether there are organ specific responses induced by tumor derived exosomes.

After long-term injection in vivo, exosomes derived MKN-45 and MKN-28 impaired the immune cell subset, created a favorable microenvironment that promoted metastasis formation in the lung. The continuous accumulation and uptake of MKN-45 and MKN-28 derived exosomes in the lung formed a immunosuppressive microenvironment by accumulating MDSC and Treg cells^41^, while decreasing NK and CD8^+^ T cell frequency. Metastatic lesions were found in mice exposed to exosomes derived from all three gastric cancer cell lines. MKN-45 derived from undifferentiated carcinomas, had natures of not only ordinary gastric mucosa but also intestinal metaplastic mucosa. It seems to have multipotentiality for differentiation, and preserved well the natures for long periods of culture^42^. This maybe the reason why MKN-45 derived exosome function is more than other two cancer cells derived exosome. MDSC are a heterogeneous population of immature myeloid cells with immunosuppressive activity that are expended in states of cancer and are associated with disease progression and poor prognosis^43^. Tumor derived exosomes induced MDSCs polarizing monocyte into highly expressing CD163-M2 phenotype, along with promoting tumor microenvironment formation and accelerating Th2 immune response^44^. Tumor derived exosomes promoted Treg proliferation effectively inducing immune suppression^41^. The present study performed a comprehensive imaging analysis to show the lung specific effects of exosomes from different differentiation status gastric cancer on immune content and its ability to create a premetastatic niche capable of metastatic colonization. Our findings provide a key evidence that gastric cancer derived exoxomes have an important function in promoting metastasis through manipulation of the immune system response.

It was well documented that exosomes contain a unique repertoire of mRNAs, miRNAs and proteins^45–48^. miRNAs can be transferred from exosomes of tumor cells to immune cells to affect gene expression and cell behavior^31,49^. Exosomal miR-451 actes as an indicator for poor prognosis of gastric cancer patients and through trigging mTOR affected Th17 differentiation in gastric cancer tissue^50^. Another study found that tumor derived exosomal protein galectin-1 induced a suppressor phenotype and may be a potential therapeutic target to prevent T cell dysfunction and enhance anti-tumor immune responses^24^. Future studies are clearly needed to idenfity the content of exosomes from gastric cancer, to define the key effector(s) and mechanisms that directly contribute to immune suppression and metastasis.

In summary, we show that gastric cancer exosomes produce an immunosuppressive microenvironment through decreasing CD8^+^ T cell and NK cell number, increasing CD4^+^T cell number and recruiting MDSC. CD8^+^ T cells are the central immune cell to attack the tumor and we provide experimental evidence that tumor derived exosome changed gene expression and cytokine secretion patterns which might be associated with the enrichment of the contents in tumor derived exosome. Cancer cells release exosomes at an elevated level and their cargo is important for cancer progression. Given that tumor derived exosome alter the composition and induce an immunosuppressive microenvironment by educating immune cells and owning to the complexity and heterogeneity of gastric cancer, elucidation of the specific biologic and molecular mechanisms leading to immune escape of tumor derived exosome and its contents (protein and RNA) in vitro and in vivo will be important to develop future novel therapeutic intervention strategies for combating gastric cancer metastasis.

## Methods

### Reagents and antibodies

PBS, FBS, culture medium (RPMI1640 and DMEM) were purchased from Gibco (ThermoFisher Scientific, USA). Anti CD8 antibody and apoptosis kit were purchased from BD Biosciences. Cyto C, Alix and Tsg101 antibodies were purchased from Cell Signaling Technologies. The ELISA kit was purchased from Quantikine. Oligo-dT and dNTP were purchased from Takapa. Reverse transcription reagents and Real-Time PCR reagents were purchased from ThermoFisher Company. Primers were synthesized by Suzhou Haoxun Biotech Co., Ltd.

### Mice

C57BL/6 mice were used at 8 weeks of age. All the animal protocols for experiments were approved by the Animal Experimental Ethics Committee of the Third Affiliated Hospital of Soochow University and carried out at the Animal Experimental Center of Soochow University. All experiments were performed in accordance with guidelines and regulations of the animal care committees of Soochow University.

### Cell lines and cell culture

Human gastric cancer cell lines (MKN-28, MKN-45 and SGC-7901) were purchased from Cell Bank of Chinese Academy of Sciences (Shanghai, China) at February, 2016. MKN-28, MKN-45 and SGC-7901 cultured in DMEM medium supplemented with 10% fetal bovine serum (FBS), 1% penicillin/streptomycin. All cell lines were authenticated by testing short tandem repaet at June, 2018 and tested for mycoplasma contamination every 3 months. All cells were cultured at 37 ℃ with 5% CO_2_.

24 h after plating, cells were washed twice with PBS and the growth media was changed to DMEM supplemented with 10% exosome depleted FBS. When cells reached 90% confluence, the supernatant was collected for exosome purification as described below.

### CD8^+^ T cell isolation and identification

PBMCs from adult healthy donors used in this study were obtained from the Third Affiliated Hospital of Sochoow University. The protocol were approved by Ethics Committee of the Third Affiliated Hospital of Soochow University [certificate No. (2016)027] and performed in accordance with the guidelines of Helsinki Declaration. The informed consent was obtained from all donors before samples collection. Immunobead-based capture was used to isolate CD8^+^ T cells from PBMC. Flow cytometry was used to measure the purity of CD8^+^ T cells.

### Exosome purification and identification

#### Purification

To collect exosomes from the cell supernatant, the conditioned medium was harvested and spun first at 300 g for 10 min to remove cell debris, and then centrifuged at 2,000 g for 10 min. The conditioned medium was then ultracentrifuged at 100,000 g for 70 min to pellet exosomes, a second washing step was performed by resuspending the precipitate in 25 ml PBS and ultracentrifuged at 100,000 g for another 70 min to obtain exosomes. The pellet was resuspended in PBS^51^. All ultracentrifugation steps were performed using the Beckerman Coulter Type 70 Ti rotor at 4 ºC. The purified exosomes were measured by bicinchoninic acid (BCA) assay kit as an indicator of the amount of exosomes isolated. The size distribution and characterization of exosome particles was detected by transmission electron microscope (TEM), the surface protein markers of exosomes were detected by Western blot.

### Exosome identification

#### TEM

Exosomes were observed, and micrograph images were taken by TEM. Samples were placed on a formvar coated copper plate for 2 min, rinsed with ultra-pure water, and 1% uranyl acetate (UA) dye with negative staining. UA was removed and the grid was washed with 15 μl of PBS for 1 min. The grid was blotted dry and left to air-dry for 15 min. Then the samples were observed by TEM (Tenia T12).

#### Western blot

Exosomes were lysed in RIPA buffer for protein extraction. Protein concentrations were determined using a BCA protein assay kit. Protein samples were separated by 10% SDS‐PAGE and then transferred to PVDF membranes. The membranes were incubated with the indicated primary antibodies with TBST containing 5% non-fat milk at 4 °C overnight, then with secondary antibodies conjugated to horseradish peroxidase at room temperature for 1 h. Finally, the membranes were incubated with ECL reagents for 4 min. The membranes were examined using a chemiluminescence photo documentation system photographed and quantitated^52^.

### Apoptosis assay

Cell apoptosis was measured using Annexin V‐FITC kit from BD Bioscience. After incubation with exosomes from three cell lines derived at different concentrations (1, 10, 100 μg/ml) for 48 h, CD8^+^ T cells were harvested and washed twice with PBS. The cell pellets were resuspended in binding buffer, and Annexin V‐FITC buffer and PI staining solution were added into the mixture and incubated at room temperature for 15 min in the dark. Additional binding buffer was added, then cell apoptosis was measured by flow cytometry (FACS Aria III, BD BioSciences). The results were analyzed by FlowJo software version 10.5(https://www.flowjo.com).

### Cell cycle assay

Cell cycle assays were performed by flow cytometry. After co-incubated with 1, 10, 100 μg/mL of MKN-28, MKN-45 or SGC-7901 derived exosomes for 48 h, CD8^+^ T cells were harvested and fixed with 75% ethanol at 4 °C for 24 h, followed by staining in PI solution for 30 min at 4 °C. CD8^+^ T cell cycle stage was determined by flow cytometry. The results were analyzed by FlowJo software version 10.5.

### Real-time PCR

Following the incubation of CD8^+^ T cells with exosomes derived from MKN-28, MKN-45 or SGC-7901 at 100 μg/ml concentration for 48 h, total RNA was extracted using TRIzol. mRNA expression was measured using SYBR Green Real-Time PCR. The expression of GADPH was used for normalization, the sequences of the forward and reverse primers used are presented in Supplementary Table S1.

### Cytokine assay

CD8^+^ T cells were stimulated with exosomes derived from MKN-28, MKN-45 or SGC-7901 cells at 100 μg/ml for 48 h, and cytokine production was measured by ELISA kit based on the manufacturer’s instructions.

### DiD-labeled exosomes

Purified exosomes were labeled with DiD according to the DiD kit instructions. Briefly, DiD reagent was diluted at 1:1,000. Exosomes were incubated with it for 10 min. After washing with 20 ml of PBS, centrifugation at 100,000 g for 70 min was performed to obtain DiD labeled exosomes.

### Imaging and tracking analysis in vivo

Three labeled gastric cancer cells exosomes were injected into the mice via tail vein (20 μg/mice), and the distribution of exosomes in each organ was observed at 4 h and 48 h after injection. the distribution of exosomes in the bone marrow, spleen and lung tissue were observed by fluorescence imaging system (PerkinElmer).

### Pre-metastasis niche microenvironment formation

Exosomes derived from gastric cancer cell lines were injected into mice (20 μg/mice) via tail vein, every 5 days for 30 days, and the volume of exosome injected into mice keep the same according to the previous concentration of protein of exosome. Bone marrow, spleen and lung tissues were harvested after injection. The immune cell subsets in the bone marrow, spleen and lung were analyzed by flow cytometry. PBS injection was used as a control group.

### Metastasis site formation study

Three gastric cancer exosomes were injected into mice via tail vein (20 μg/mouse) every 5 days for 30 days, mouse received MFC cells (1 × 10^5^/mouse) via intravenous injection on day 30, lung tissues were collected on day 60, tumor formation was assessed via hematoxylin and eosin staining to evaluate metastatic formaton in the lung.

### Statistical methods

Cell culture, ELISA and real-time PCR experiments were performed in triplicate. Statistical analyses of the data were performed using SPSS 21.0 version (SPSS Inc, Chicago, IL). Graphs were generated using GraphPad Prism (version 6.0, GraphPad software Inc., San Diego, California, USA, https://www.graphpad.com). Quantitative data are presented as the mean ± standard error of mean (mean ± SEM). Non-parametric data were analyzed by two-tail Mann–Whitney *U* tests. Parametric data were analyzed by ANOVA. Differences with *P* values < 0.05 were considered significant.

## Supplementary information

Supplementary Information.
