# Supplementary material for: Immune suppressed tumor microenvironment by exosomes derived from gastric cancer cells via modulating immune functions
Source: Sci Rep. 2020 Sep 8;10:14749. doi: 10.1038/s41598-020-71573-y (PMC7479614; doi:10.1038/s41598-020-71573-y)
Supplement: Supplementary file 1 — Supplementary Information. [file 41598_2020_71573_MOESM1_ESM.docx]

**Supplementary material**

**Immune Suppressed Tumor Microenvironment by Exosomes Derived from Gastric Cancer Cells via Modulating Immune Functions**

Juan Liu^1,2,3, 4^, Shaoxian Wu^1,2,3^, Xiao Zheng^1,2,3^, Panpan Zheng^1^, Yuanyuan Fu^1^, Changping Wu^1^, Binfeng Lu^5^, Jingfang Ju^4^, Jingting Jiang^1,2,3^

^1^Department of Tumor Biological Treatment, The Third Affiliated Hospital of Soochow University, Changzhou 213003, China.

^2^Jiangsu Engineering Research Center for Tumor Immunotherapy Changzhou 213003, China.

^3^Institute of Cell Therapy, Soochow University, Changzhou 213003, China.

^4^Department of Pathology, Stony Brook University, New York 11794, US.

^5^Department of Immunology, University of Pittsburgh, Pittsburgh 15213, US.

**Supplementary material:**

full-length blots of figure 1B
